# Editorial: Musculoskeletal Adaptations to Training and Sports Performance: Connecting Theory and Practice

**DOI:** 10.3389/fphys.2022.866895

**Published:** 2022-03-03

**Authors:** Daniel A. Marinho, Ricardo Ferraz, Argyris G. Toubekis, Henrique P. Neiva

**Affiliations:** ^1^Department of Sport Sciences, University of Beira Interior, Covilhã, Portugal; ^2^Research Center in Sports Sciences, Health Sciences and Human Development, CIDESD, Covilhã, Portugal; ^3^School of Physical Education and Sports Science, National and Kapodistrian University of Athens, Athens, Greece

**Keywords:** sports, performance, physiology, biomechanics, motor strategies

We are pleased to present the Research Topic on Musculoskeletal Adaptations to Training and Sports Performance: Connecting Theory and Practice. With this Research Topic, we intend to increase knowledge on musculoskeletal adaptations to training with a strong focus on the connection between training practices and sports performance. To improve sports performance, coaches and researchers must better understand the training process, the dose-response relationship, and adaptations to training load. The scope of this research ranges from the general discussion of trainability and athlete responses to new assessment tools commonly used in other areas of investigation (e.g., medicine).

The adaptive process to training is discussed by Radak and Taylor and is focused on the specificity of trainability. The authors highlight some issues on the systemic nature of exercise-induced adaptation, alerting that the common use of the term “non-responders” could hide the view of a real specific response. The fact that a specific physiological function or system does not reveal positive changes to a training program does not mean that all other functions/systems will not respond positively. Thus, we should be aware of the specificity of the analysis that is performed and highlight one of the most valuable principles of training, which is specificity. The specificity of training responses can be influenced by preparatory activities (i.e., warm-up) and recovery mode (i.e., cooldown). Since early on, the sports community has been aware of the key role of warm-up and the literature has related the benefits of warm-up with changes in the physiological status of the athletes. Coaches and athletes have used different tasks during warm-up including dynamic and static stretching exercises, believing that these can improve performance later on. Moreover, it is suggested that some types of stretching may reduce muscle stiffness and increase the range of motion, thus decreasing the incidence of activity-related injuries. However, some discussion emerged in the literature when it was found that there is a loss of strength and power performance following stretching tasks. The opinion article by Afonso, Olivares-Jabalera, et al. debates the use of stretching during warm-up. Furthermore, the authors go further on the discussion about stretching, raising relevant issues such as “*Can I” Vs. “Do I Have” To Stretch in the Cool-Down Phase?; “Can I” Vs. “Do I Have To” Stretch To Chronically Improve Range of Motion? “Can I” Vs. “Do I Have To” Stretch To Reduce Injury Risk?* These questions create a link to other contributions to this Research Topic, such as the review presented by Afonso, Clemente, et al. In this review, the authors analyzed and discussed the effects of post-exercise stretching on muscle recovery markers (e.g., the range of motion and onset muscular soreness). Curiously, more than 70% of the analyzed studies comparing passive recovery (i.e., rest) and stretching found no differences for recovery markers. Stretching is usually recommended to enhance post-exercise recovery but the data is too scarce and heterogeneous to support guidelines.

Research on injury risk goes beyond questions about stretching exercises. This is clearly described by Afonso, Rocha-Rodrigues, et al. providing deeper insight into the individual risk factors for hamstring injuries. The authors presented an interesting perspective of the risk of injury based on modifiable factors (e.g., warm-up, load, fatigue, lumbopelvic hip stability, motor patterns, cardiovascular fitness, mobility, lower back pain, recovery strategies, strength, asymmetry, nutrition, and psychosocial factors), and non-modifiable factors (e.g., age, previous injury, specific anatomic variations). Physical training programs should be developed to mitigate the risk of injury with a focus on modifiable factors but keeping in mind that there is a high level of anatomic variability among different subjects, thus highlighting the need for better-individualized exercise interventions. In addition to being an influencing factor for training design, the anatomical characteristics of the athlete have been increasingly recognized as influencing performance in different sports. For instance, the architecture of gastrocnemius medialis and gastrocnemius lateralis is different between female cyclists and basketballers (May et al.). Some previous literature supports that these differences could be explained by the different stimulus that the gastrocnemius muscle is subjected to in the typical gesture performed in each sport. However, in this study, it appears that the distinct mechanical stimuli caused by cycling and basketball training did not influence gastrocnemius muscle architecture in male athletes. This emphasizes that there is still much to be understood about the anatomical and physiological adaptations to training and their relationship with performance. For example, contrary to expectations, Ando et al. found that 8 weeks of drop jump training resulted in decreased passive stiffness of the medial gastrocnemius. Nevertheless, drop jump performance was improved. Once again, this underlines the specificity of adaptations to training in each sport.

Research in sports science has been increasingly aware that different sports require different stimulations and, consequently, adaptations should also be different. The impact of the sport, different training methods, and evaluation tools have not been deeply explored in this Research Topic. However, some examples are provided. Specific strength training programs were developed for dancers (see Ávelia-Carvalho et al.) and young soccer players (see Falces-Prieto et al.) resulting in better jumping performance, aerobic endurance, and body composition. New training methods were explored, such as the combination of sprint interval exercises in hypoxia or with blood flow restriction (see Solsona et al.), and the use of new evaluation tools, such as arterial doppler ultrasound to analyze the arterial and venous diameters of the lower limbs in indoor soccer athletes (see Mateus et al.).

We strongly believe that increasing understanding of the adaptations to the exercise, from physiological, to biochemical–molecular, to structural-anatomical, should be a core aspect of sports science research to provide background knowledge to support coaches and athletes in their activities and, thus, enhance performance. Here emerges one of the most recent challenges for sport-related professionals, which is the translation of theoretical content and scientific findings into a practical setting.

## Author Contributions

All authors listed have made a substantial, direct, and intellectual contribution to the work and approved it for publication.

## Funding

This work was supported by the Portuguese Foundation for Science and Technology (FCT), I.P., under project UIDB04045/2020.

## Conflict of Interest

The authors declare that the research was conducted in the absence of any commercial or financial relationships that could be construed as a potential conflict of interest.

## Publisher's Note

All claims expressed in this article are solely those of the authors and do not necessarily represent those of their affiliated organizations, or those of the publisher, the editors and the reviewers. Any product that may be evaluated in this article, or claim that may be made by its manufacturer, is not guaranteed or endorsed by the publisher.

